# Population Dynamics and Tree Damage of the Invasive Chestnut Gall Wasp, *Dryocosmus kuriphilus*, in Its Southernmost European Distributional Range

**DOI:** 10.3390/insects12100900

**Published:** 2021-10-02

**Authors:** Javier Quinto, María Eva Wong, Juan Ramón Boyero, José Miguel Vela, Martin Aguirrebengoa

**Affiliations:** Centro IFAPA de Málaga, Laboratorio de Entomología Agrícola, Instituto de Investigación y Formación Agraria, Pesquera, Alimentaria y de la Producción Ecológica, 29140 Málaga, Spain; mariae.wong@juntadeandalucia.es (M.E.W.); juanr.boyero@juntadeandalucia.es (J.R.B.); josem.vela@juntadeandalucia.es (J.M.V.); martin.aguirrebengoa@juntadeandalucia.es (M.A.)

**Keywords:** abiotic factors, biological invasions, *Castanea sativa*, Cynipidae, forest pest, pest management, temperature, *Torymus sinensis*

## Abstract

**Simple Summary:**

Chestnut cultivation makes it possible to invigorate the economy of many rural areas in Europe. The chestnut gall wasp *Dryocosmus kuriphilus* is a serious invasive pest that causes severe damage to chestnut cultivation worldwide. Its rapid spread across Europe endangers the continuity of the entire chestnut industry. Despite this growing concern, scarce attention has been paid to the status of *D. kuriphilus* in its southernmost distributional range in continental Europe and limited knowledge on the factors modelling their populations is available. In this study, we assessed spatio-temporal patterns in the population dynamics, phenology and tree damage in southern Spain, and further evaluated the relationship between these variables and thermal trends. Strong variation in the population dynamics and flight phenology was found both among localities and over time, which was influenced by differences in thermal regimes. Similarly, tree damage evolved differently over time in each locality, thus suggesting that local conditions may determine significant differences in damage evolution. Our work contributes to a better understanding of this pest in countries throughout the Mediterranean basin and can be useful for further improvement of control and management strategies.

**Abstract:**

The invasive chestnut gall wasp (CGW), *Dryocosmus kuriphilus*, the worst pest of chestnut cultivation, has spread worryingly throughout Europe in less than 20 years. Despite the great concern around this pest, little is known about the status in its southernmost distribution in continental Europe. We assessed spatio-temporal patterns in the population dynamics, phenology and tree damage caused by CGW in southern Spain. Likewise, the relationship between these variables and thermal trends was evaluated. We found strong variation in the population dynamics and flight phenology among localities and over time, which were highly influenced by changes in thermal regimes. Specifically, warmer localities and vegetative periods promoted higher population densities, a partial increase in the survival of immature stages, and advanced flight activity. Moreover, tree damage evolved differently over time in each locality, which suggests that local conditions may determine differences in damage evolution. Our findings evidence that great spatio-temporal variability in the CGW populations takes place across invaded areas in its southernmost European distributional range. Although control mechanisms have been introduced, implementation of further control and management measures are critical to cope with this main threat for the chestnut industry and to prevent its spread to nearing chestnut-producing areas.

## 1. Introduction

Biological invasions determine multiple pervasive effects on ecosystem processes and functions [1,2]. They also cause huge economic impacts, such as direct production losses in agricultural/forestry systems and related management costs [3,4]. Such impacts may substantially differ between invasive species and contexts [5,6,7]. For instance, invasive phytophagous species associated with woody plants usually show higher spread rates and potential for invasiveness than those associated with herbaceous plants [8]. Likewise, spatio-temporal differences in environmental characteristics in forest systems determine changes in population growth and the rate of spread of invasive forest insects [9]. Studies assessing invasion spread patterns and underlying processes are essential to understanding, predicting and managing invasions.

The chestnut gall wasp (CGW) *Dryocosmus kuriphilus* Yasumatsu (Hymenoptera: Cynipidae) is a parthenogenetic gall-former considered to be the most serious pest of chestnuts (*Castanea* spp.) worldwide [10]. The sweet chestnut (*Castanea sativa* Mill.) is the only native member of *Castanea* genus in Europe, where it covers an area of approximately 2.5 million ha [11]. Chestnut cultivation for fruit and timber production promoted the spread of this deciduous tree species throughout the continent after the Roman period, so the present distribution is in part the result of that broad dissemination [12]. This fact resulted in the establishment of this tree species at the limits of its potential ecological range in many European mountain areas. The sweet chestnut grows well on limestone-free and highly weathered (acidic to neutral) soils, with optimal annual rainfall ranging from 400 to 1600 mm and optimal annual mean temperature between 8 °C and 15 °C [11,13]. The earliest detection of CGW in Europe occurred in northwestern Italy in 2002 [14], and subsequently in 19 other countries [10]. The transport of plant material from infested areas has likely driven this rapid spread throughout much of the continent [15,16]. The first records in Spain took place in the region of Catalonia in 2012 [17], and currently, only certain areas in central, western and southwestern continental Spain remain free of this major invasive pest [16].

CGW is a univoltine species highly synchronized with the vegetative period of chestnut trees [18,19,20,21], existing a close relationship between their potential fecundity and the availability of suitable buds [22,23]. The egg hatching takes place approximately one month after the egg-laying, and the first instar larvae enter diapause over one month later and overwinter in the newly infested buds until the next spring bud burst. Once it happens, the larvae continue their immature development. Larval feeding induces the formation of characteristic greenish to reddish and globular in shape galls, inside which the CGW completes its development and from where sexually adult females will emerge from the late spring to the early summer (according to studies performed in Italy and northern Spain, e.g., [20,21,24]). In the early stages of the CGW invasion (under low population pressure), the adults tend to lay fewer eggs per bud because this induces the formation of small uni-lobed galls, a fact that is associated with increased fecundity of emerging females [22]. As the population density increases, the number of eggs per bud becomes higher and larger and multi-lobed galls are formed [21,25,26,27].

The formation of numerous galls blocks the normal flush movement, which implies a reduction of tree vigor and tree-ring growth, the alteration of the branch architecture and the inhibition of the development of new shoots [28,29,30,31,32,33]. Successive severe attacks may lead to reductions in the total leaf area greater than 70% [31], which cause a drastic decrease in photosynthetic capacity and in biomass production [29,30,34], ultimately resulting in fruit yield losses that may exceed 80% [35]. However, even more concerning is the fact that high levels of infestation over time lead to a reduction in the number of dormant buds, thereby affecting the regeneration capacity of the trees [31,36] and jeopardizing the continuity of the entire chestnut industry.

Differential environmental conditions can result in relevant spatial and temporal variability in the phenology of the CGW. For instance, in warmer and drier years the egg hatching occurs earlier [24], and the immature development is shortened at higher seasonal temperatures [19,37,38], frequently involving higher population densities. Moreover, the duration of the immature development is positively related to the altitude and latitude of chestnut cultivation, in particular, to lower mean temperatures [39]. The annual mean temperature also affects the gall mass and volume, and larger galls are formed in chestnuts in the Mediterranean biogeographical region [40]. According to the microenvironment hypothesis, galls confer protection to insects against extreme climatic events as desiccation, ultraviolet radiation, frosts, or severe droughts [41,42,43,44], in this way facilitating the spread and establishment of gall-inducing insects in new areas. On the other hand, the CGW adult activity is favored under optimal temperatures between 25–30 °C, decreases with temperatures below 15 °C and is virtually inexistent below 10 °C [45]. Given this close dependence on temperature regimes, chestnuts in southern Spain are likely to be particularly suitable for CGW, as predicted by Gil-Tapetado et al. [16,40]. This could help to explain why its distributional range rapidly increased throughout the southern Spanish province of Málaga [46]. It is therefore entirely appropriate to carry out studies assessing spatio-temporal patterns in population dynamics for control and management purposes.

In this study, we aimed to (a) assess spatio-temporal variability in the population dynamics and tree damage of the invasive CGW in the southernmost chestnuts in continental Europe (namely southern Spain), and (b) address the relationship between these variables and fine-scale temperature patterns. Considering the high suitability and susceptibility for colonization by CGW across the Mediterranean region [16,37,40,47], we predicted high and similar infestation rates, population dynamics and levels of damage across recently invaded areas in southern Spain. Moreover, the previously documented relationship between thermal regimes and CGW population dynamics [16,37,39] led us to expect the existence of differences within and among years across the study area. Our final goal was to provide information about the status of this major pest to implement efficient control and management measures.

## 2. Materials and Methods

### 2.1. Chestnut Cultivation in the Study Area

**Figure 1:**
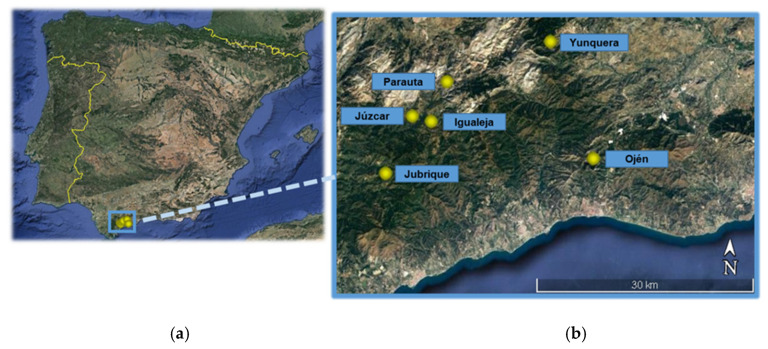
Orthoimage showing the location of the six sampled chestnut orchards in southern Spain (**a**) and within the province of Málaga (**b**).

The southernmost sweet chestnut orchards of mainland Europe are in Andalusia (southern Spain). Chestnut cultivation covers about 9000 ha in Andalusia, a third of which takes place in the province of Málaga [48], mainly traditional agricultural systems in the upper basin of the River Genal and in the foothills of the Sierra de las Nieves (approximately 36 30’–36 50’ N, 4 50’–5 20’ W; see Table 1 and Figure 1 for details of the selected localities within this area). It is also worth noting that sweet chestnut woods (Mediterranean *Castanea sativa*-dominated forests and old-established plantations with semi-natural undergrowth) are listed in Annex I of the EU Habitats Directive, so this species-rich ecosystem entails high conservation priority [49]. Local threats to chestnut production are the ink disease (*Phythophtora* spp.), the canker blight (*Chryphonectria parasitica*), the chestnut tortrix (*Cydia splendana*) and, in particular, the invasive non-native CGW [46,50]. The first detections of CGW in Andalusian chestnut orchards occurred in Málaga in 2014, specifically in the municipality of Ojén, from where it spread to the rest of the studied localities in only one year. Since 2016 its distributional range and population density dramatically increased, and important outbreaks can nowadays be found in the main chestnut-producing areas [46], thus becoming a serious threat to this traditional source of income for local people.

### 2.2. Life-Cycle Parameters

Descriptive studies about the CGW life-cycle parameters under field conditions are uncommon in the literature (but see [51]). We collected 2510 chestnut-infested buds–galls from four localities during two consecutive vegetative periods, 2016–2017 and 2017–2018 (Table 1). The sampling unit was the bud–galls, i.e., a bud before sprouting, and posteriorly the total number of galls coming from a single infested bud after sprouting. Fourteen samplings between 3 November 2016and 11 July 2017were carried out for the 2016 CGW cohort (monthly from November to February, and every 15 days from March), while eleven samplings between 26 July 2017 and 1 August 2018 were done for the 2017 CGW cohort (monthly from July to May, and every 15 days from June). In the first sampling dates, non-infested buds (those without eggs) were not considered. Sampling ended in mid-July to early August in both vegetative periods, when most adults had already emerged. We collected 20 buds–galls per locality on average in the samplings done in 2016–2017, and 26 buds–galls per locality on average in 2017–2018. At the lab, the buds–galls (hereafter simply buds) were dissected and the number of CGW eggs, larvae (L1, L2 and L3), pupae and adults at each bud were counted, following Viggiani and Nugnes [20]. A total of 3808 larvae were obtained through bud–gall dissections, 1120 of which were mounted to confirm larval stages under microscope. With these counts we calculated (a) the average development stage of CGW individuals in each of the samplings in each of the localities per cohort, (b) the average number of CGW individuals per development stage and per bud in each of the localities per cohort, and (c) the average survival probability per stage and in total (egg-to-adult) in each of the localities per cohort.

In addition, we used age-specific life-tables to calculate the following population parameters of CGW:

(a) The net reproduction rate (*R*_0_):

*R*_0_ = ∑ l_x_ × b_x_, (1)

where l_x_ is the percent of the original cohort that survives to age/stage x, and b_x_ is the number of offspring produced per individual in age/stage x. The results of Equation (1) can be interpreted as follows: *R*_0_ < 1 indicates the members of the population are not replacing themselves (i.e., the population is declining), *R*_0_ > 1 denotes an increasing population, and *R*_0_ = 1 indicates a stationary or “stable” population);

(b) The intrinsic rate of increase (*r*):

*r* = ln *R*_0_/*T*, (2)

where *T* is the generation time (the time between the birth of one cohort and the birth of their offspring). The results of Equation (2) can be interpreted as follows: *r* > 0 indicates an increasing population, *r* = 0 indicates a stationary population, and *r* < 0 indicates a declining population, and

(c) The finite rate of population change (*λ*):

*λ* = *N_t+_*_1/_*N_t_*, (3)

where *N_t_* is the population size at time t and *N_t+_*_1_ is the number of individuals after one time period (around one year in our case). The results of Equation (3) can be interpreted as follows: *λ* > 1 indicates an increasing population, *λ* = 1 indicates a stationary population, and *λ* < 1 indicates a decreasing population. Life-table analyses are useful to describe development times and survival rates for a population, and therefore to estimate rates of population changes [52]. It should be noted that life-tables were calculated under variable thermal regimes, as the bud–gall sampling was performed in natural areas.

### 2.3. Flight Phenology

We studied the flight phenology of CGW during four years (2017–2020) in six localities (Table 1). We placed two yellow 22 × 18 cm adhesive traps in five randomly selected trees per locality, which were replaced every three to four days (mean ± SE = 3.72 ± 0.06; median = 4) from June to August each year. This kind of sampling has proven effective for assessing the adult flight period of CGW [21]. We then calculated the average number of CGW individuals per trap and day and the total number of CGW individuals per trap and year.

### 2.4. Tree Damage

We assessed temporal changes in the impact of CGW on chestnut orchards in four localities from 2017 to 2020 (2015–2020 in the case of Ojén) (Table 1). The sampling was focused on chestnut sprouts produced in the previous vegetative period with respect to sampling date, namely the most frequently attacked vegetative organ [29], therefore representing a useful indicator of the level of infestation. Previously sampled trees (namely those used to assess juvenile stages) were excluded for the random selection of sprouts. A total of 3094 sprouts were collected, an average of 15.50 ± 0.75 sprouts per tree/locality/year (median = 14). In all years, the collection of sprouts was done in mid-autumn (October-November). The number of galls per sprout was recorded, and (a) the average number of sprouts with CGW galls per tree, and (b) the number of CGW galls per sprout per tree were calculated.

### 2.5. Temperature Data Calculation

We obtained fine-scale spatio-temporal temperature values for each sampling date in each of the localities. For this purpose, we first obtained the historical monthly and annual mean temperature for each of the localities from the 1971–2000 Iberian Climatic Atlas of AEMET (State Meteorological Agency of Spain) [53], which provides georeferenced data with an accuracy of one km^2^. Next, we obtained the daily mean temperature values for the 2015-2020 period of the closest and most accurate weather station of AEMET (Ronda 6032X) [54], as well as the historical monthly and annual mean temperature of this exact point from the 1971-2000 Iberian Climatic Atlas. We estimated the mean daily temperature for each locality in the period 2015-2020 by comparing the variation of historical values of monthly mean temperatures of each locality with respect to the Ronda 6032X meteorological station. To calculate the cumulative °C in life-cycle parameters and flight phenology (the sum of daily mean °C on a specific date) for each locality in each season, we initiated the count per locality at the exact moment of CGW flight peaked the previous season. In the case of total CGW individuals per trap and season, the cumulative °C per season were summed until the end of each season’s flight. In the case of chestnut damage measures, annual mean temperature per locality was considered.

### 2.6. Statistical Analyses

General Linear Models (GLMs) were performed to test the effects of year and locality on all measured variables. All variables were analyzed with GLMs after checking their error distributions and homoscedasticity compliance. In the case of life-cycle parameters and flight phenology, sampling date or temperature were also considered as explanatory variables (such variables were never analyzed together because of their high collinearity), whereas annual mean temperature was considered to evaluate tree damage. In the case of the number of CGW individuals across developmental stages/bud and sampling, a multiple response GLM was used (number of CGW eggs, L1, L2, L3, pupae and adults per bud as the six dependent variables of the same model). We performed separate analyses per season because of the high variability in the number and periodicity of samplings. In the case of the average number of CGW individuals per developmental stage and bud, and the average survival probability per developmental stage, locality × year interaction was not considered due to lack of required residual degrees of freedom. In the case of the average number of CGW flying individuals per trap and day, GLMs with sampling date (or cumulative °C) × locality × year were performed. In the case of the total number of CGW individuals per trap and year, a GLM with cumulative °C × locality and cumulative °C × year as explanatory variables was carried out. The lack of required residual degrees of freedom impeded testing cumulative °C × locality × year triple interaction effects, and even locality × year effects. In the case of chestnut damage variables, two models were carried out: one was year × locality, and the other was annual mean temperature × locality + annual mean temperature × year. When factor (locality, year) or interaction level effects were significant, Tukey’s HSD pairwise post hoc comparisons were performed to determine significant differences between levels of each factor. All analyses were performed with R version 3.5.2 [55]. GLMs were performed using the package nlme [56], explained variance was calculated using the rsquared function of the piecewiseSEM package [57] and Tukey’s HSD pairwise post hoc comparisons were performed using the emmeans package [58].

## 3. Results

### 3.1. Life-Cycle Stages and Population Trends

**Figure 2:**
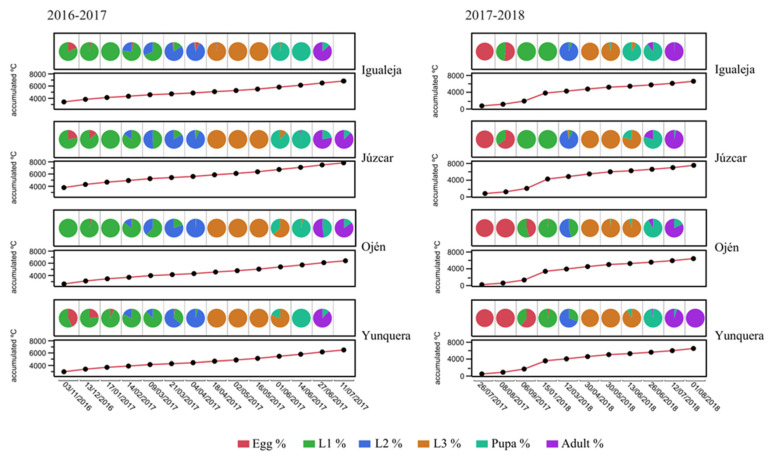
Variation in the relative abundance of CGW development stages inside chestnut buds–galls during the two assessed consecutive vegetative periods. Empty cells denote that all adults had already emerged from the galls. Below pie charts, the cumulative °C in each of the samplings in each locality is shown.

In the 2016 cohort, sampling date but not locality nor their interaction influenced the average number of CGW individuals across developmental stages and per bud (Table 2, see a relative abundance of each development stage per sampling in Figure 2). When cumulative °C was considered instead of sampling date in the 2016 cohort, the locality had a significant effect on the average number of CGW individuals across developmental stages and per bud (the warmest locality, Júzcar, differed from the rest of localities, Table S1), and explained variance increased (Table 2). In the 2017 cohort, sampling date (or cumulative °C) × locality had a significant effect on CGW individuals across developmental stages and per bud, and explained more variance than in the 2016 cohort.

**Figure 3:**
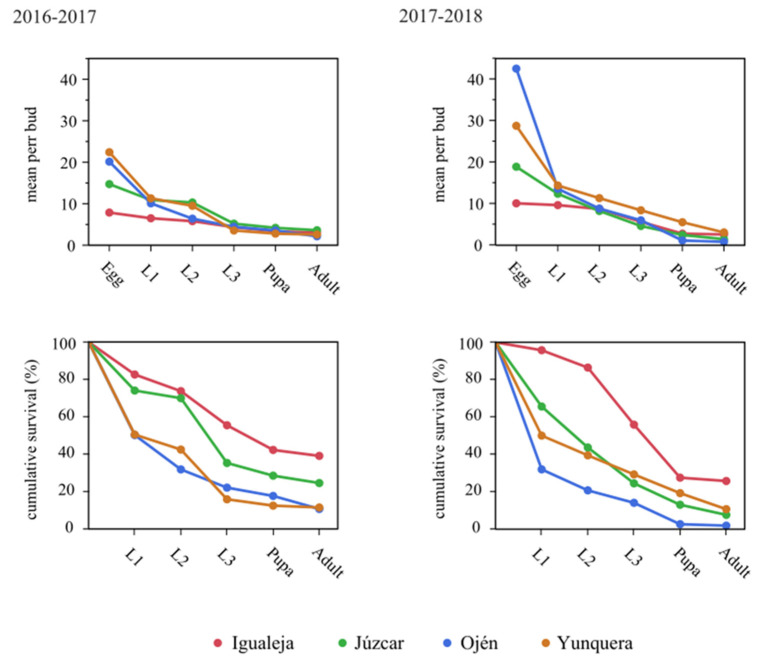
Pre-flight CGW relative abundance and survival in the sampled localities of Málaga. Above, mean number of each development stage of CGW per chestnut buds–galls in each locality at each vegetative period. Below, average survival probability per development stage in each locality at each vegetative period. See locality × year clustered heatmap in Figure S1.

With regard to the effect of locality and vegetative period on the average number of CGW individuals per development stage and per bud, both locality (*F*_3,3_ = 21.66, *p* = 0.015) and vegetative period (*F*_1,3_ = 39.62, *p* = 0.008) had a significant effect on L1/bud (Figure 3). That is, L1/bud number differences across localities were constant in both periods (the coldest locality, Igualeja, had less L1/bud than the rest of localities, Table S1), and L1/bud number differences across periods were constant between localities (increment in 2017-2018 with respect to 2016–2017 was similar in all localities, Table S1). Locality and period did not affect the average number of CGW individuals per bud on the other development stages (Figure 3). With respect to the average survival probability per development stage, locality had a significant effect on egg-to-L1 transition (*F*_3,3_ = 10.47, *p* = 0.042, Figure 3). So that, differences in egg-to-L1 survival across localities were constant in both periods (higher values in Igualeja with respect to Ojén, Table S1). Locality and vegetative period did not affect the average survival probability on the other development stages, although a marginally significant trend of period affecting L3-to-pupae transition was observed (*F*_1,3_ = 9.84, *p* = 0.051, Figure 3). Total average survival probability, that is, egg-to-adult survival, was significantly affected by locality (*F*_3,3_ = 10.69, *p* = 0.041, Figure 3), and marginally affected by period (*F*_1,3_ = 8.16, *p* = 0.064, Figure 3). Differences across localities on total cumulative survival were thus constant in both vegetative periods (higher values were found in Igualeja with respect to Ojén, Table S1).

The assessed population parameters revealed differences between CGW cohorts of 2016 and 2017. Ojén was the only locality showing declining populations in 2017 with respect to 2016 (2016: *R*_0_ = 2.22, *r* = 0.35, *λ* = 1.41; 2017: *R*_0_ = 0.81, *r* = -0.09, *λ* = 0.91), whereas the populations in Igualeja, Júzcar and Yunquera clearly increased in the same period (in all cases values of both *R*_0_ and *λ* > 1 and values of *r* > 0).

### 3.2. Factors Influencing Flight Phenology

**Figure 4:**
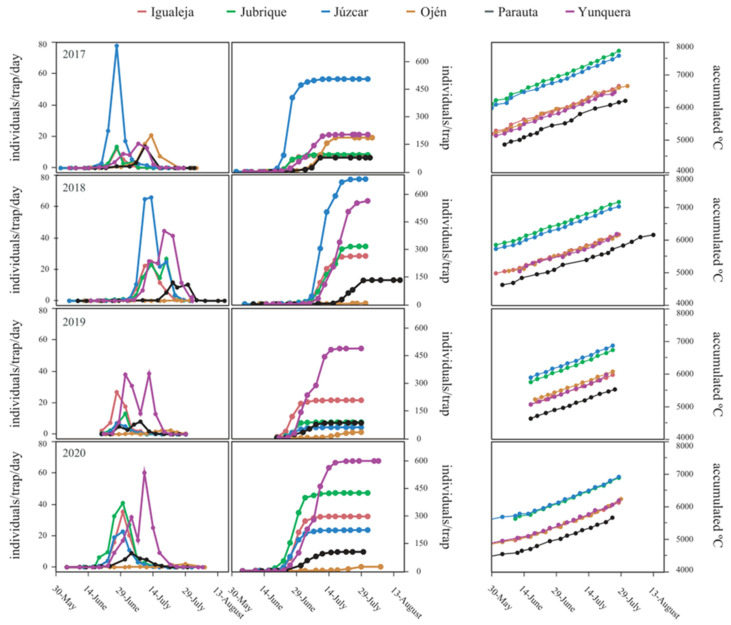
Variation in the flight period of CGW over years in the sampled localities of Málaga. On the left, line chart of year x locality effects on the number of CGW individuals per trap and day. In the middle, cumulative number of CGW individuals per trap and season. On the right, cumulative °C per sampling date, locality and season. See the effect of locality × year in the clustered heatmap for CGW flight start date, flight peak date and flight end date in Figure S2.

The flight of CGW occurred from late spring to mid-summer during 2017–2020 (the earliest flight start was on 1 June in 2017 in Júzcar, Jubrique and Igualeja; the latest flight end was on 16 August in 2018 in Parauta). The number of CGW individuals per trap and day was significantly affected by locality, as well as by the interaction sampling date × year and the interaction cumulative °C × year (Table 3, Figure 4). That is, the number of CGW individuals and their flight curve shape remained rather constant across localities in the different years (Yunquera different from Ojén and Parauta, Table S2), showing important fluctuations within each year. With respect to the density of flying CGW individuals (the sum of captured CGW individuals per trap and year), it was only significantly affected by locality (Table 3, Figure 4).

### 3.3. Damage to Chestnut Trees

**Figure 5:**
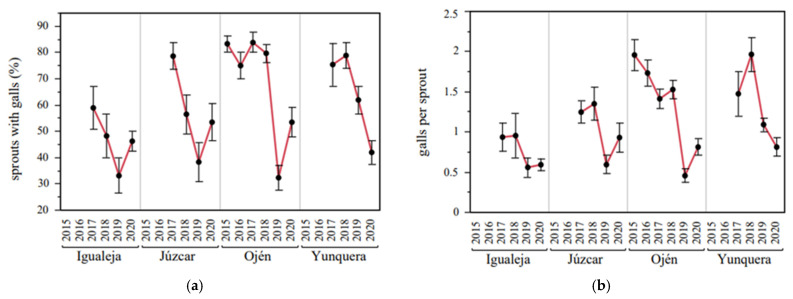
CGW damage levels across years and localities. (**a**) Year × locality effects on the rate of sprouts with CGW galls. (**b**) Year × locality effects on the number of CGW galls per sprout. See locality × year clustered heatmap in Figure S3.

The locality and the year had an interactive effect on the average number of infested sprouts (Table 4, Figure 5), since all the assessed localities showed different levels of sprout infestation and trends over the years (see significant post hoc results in in Table S3). High percentages of infested sprouts were recorded shortly after the first detections of CGW (around 60–80% in 2017), showing a considerable decrease in 2019 (around 30–40%). Moreover, the number of CGW galls per sprout was affected by locality and by year, but interactive effects were not found (Table 4, Figure 5), suggesting that differences among localities were constant over the years and vice versa (see significant post hoc results in Table S3). Finally, the annual mean temperature was not a determining factor of the damage caused, since the explained variance of the GLMs increased when this factor was not included (Table 4).

## 4. Discussion

In the present study, we found relevant spatio-temporal changes in population dynamics and tree damage of the invasive CGW across chestnut orchards in southern Spain. Moreover, the CGW population dynamics (but not tree damage) were notably influenced by variations in the annual mean temperature across localities and years. In this vein, warmer localities and vegetative periods entailed an earlier emergence and promoted higher population densities, although spatial variation in population levels was not entirely attributable to thermal regimes. Likewise, the flight phenology exhibited high spatial variation, with important fluctuations within each season. The quantitative assessment of tree damage revealed that each locality evolved differently over time; however, all of them experienced a marked decline in tree damage in less than five years. Consequently, our results were only partially consistent with our predictions since we found a contrasting spatial and temporal variation on population dynamics and tree damage. This growing knowledge base is useful for further improving control and management strategies against CGW outbreaks in southern Europe and throughout the Mediterranean basin.

Recent studies have pointed out that temperature regimes within the European Mediterranean region particularly benefit the life cycle of CGW (e.g., [16,37,40]). Environmental conditions become even more favorable in Mediterranean areas at lower latitudes [16]. Although the CGW has indeed shown great potential for colonizing chestnut orchards across Málaga [46], the population dynamics have evolved in different ways in nearby areas since the early stages of the invasion. For instance, those localities that initially had a lower proportion of eggs per bud (i.e., Igualeja, and to a lesser extent Júzcar), reached the highest average survival of adults per bud in both 2016–2017 and 2017–2018. These results may be attributed in part to spatial or temporal differences in infestation rates (as suggested by our age-specific life-tables), which very often are related to differential environmental characteristics during the development of immature stages [30,38,39,59]. In this connection, thermal trends influenced the population dynamics at both spatial (localities) and temporal scales (vegetative periods), which suggests that temperature is an important driver of density changes in CGW stages across invaded areas in southern Spain.

Temporal variations in thermal conditions also exert great influence on the emergence of the adults of CGW [21,37,60], ultimately conditioning the flight phenology. In line with similar research, the emergence in our conditions occurred earlier in warmer years [19,24,37,60]. Only 2018 broke the usual thermal trend, since it was exceptionally mild in comparison with both previous and subsequent years, a fact that usually causes a delay in the adult emergence [37]. On the other hand, the flight phenology described remarkable fluctuations across localities, but in general, the adult emergence took place earlier in warmer localities. This reinforces the idea that differences in phenology of CGW are mainly associated with the different thermal regimes recorded across study areas [39].

Damage to chestnut trees experienced an exponential increase over the first years of the CGW invasion (e.g., [30,34]). Accordingly, generalized high tree damage was recorded when our sampling started; however, all the localities underwent a marked decline in tree damage in less than five years. This highlights that the worst levels of damage in southern Spain occurred during the first two to four years. On the other hand, the striking drop in tree damage may be attributed to the action of the biological control agent *Torymus sinensis* (Hymenoptera: Torymidae), which is highly specific to CGW [61,62]. The recovery process can start earlier by shortening the time elapsed between CGW arrival and biological control by *T. sinensis* [31,36]. In our study area, a unique experimental release of *T. sinensis* was done in Ojén in 2015, while in the other localities an increasing number of annual releases have been implemented since 2016, i.e., in all cases only one year after the pest detection [46,63]. However, each locality showed a particular temporal evolution in tree damage, suggesting that the recovery process (or the establishment of *T. sinensis*) is not equivalent in all areas within a territory. Microenvironmental variables could be modeling such differences (see [40]), but further research is needed to shine a light on this issue. Interestingly enough, tree damage experienced an alarming increase in 2020 in several localities, which supports the idea that recurrent CGW infestations are expected in Mediterranean areas [62,63,64,65]. Given the rapid spatial and temporal variability found in population dynamics and tree damage, long-term studies assessing the evolution of CGW outbreaks in its southernmost European distributional range are necessary for further improvement in control and management programs.

## Figures and Tables

**Table 1:** Description of the six chestnut orchards sampled in Málaga, and the localities considered for each analysis. Historical annual mean temperatures correspond to the 1971–2000 climate records. Elevation is expressed in m a.s.l. Historical annual mean temperature is expressed in °C.

Locality	Latitude	Longitude	Elevation	Historical Annual Mean Temperature	Life-Cycle Parameters	Flight Phenology	Tree Damage
Igualeja	36.617647	−5.135904	740	13.6	✓	✓	✓
Jubrique	36.552279	−5.204078	789	15.7		✓	
Júzcar	36.623390	−5.165245	719	14.7	✓	✓	✓
Ojén	36.577863	−4.884878	812	14.2	✓	✓	✓
Parauta	36.667257	−5.113545	1049	13.1		✓	
Yunquera	36.720823	−4.954187	940	13.7	✓	✓	✓

**Table 2:** Above, multiple response GLM results showing the effects of sampling date (or cumulative °C) × locality on the number of chestnut gall wasp (CGW) per stage and bud in the 2016–2017 season. Below, the same analysis results in the 2017–2018 season.

Explanatory Variable	Individuals Across Developmental Stages and Per Bud			
	*df*	*F*	*p*	*R* ^2^
2016–2017				
Sampling date	1, 48	24.14	<0.001	0.394
Locality	3, 48	1.64	0.19	
Sampling date × Locality	3, 48	0.72	0.54	
Cumulative °C	1, 48	39.38	<0.001	0.493
Locality	3, 48	5.76	0.002	
Cumulative °C × Locality	3, 48	0.49	0.68	
2017–2018				
Sampling date	1, 36	60.32	<0.001	0.709
Locality	3, 36	3.31	0.030	
Sampling date × Locality	3, 36	5.91	0.002	
Cumulative °C	1, 36	72.12	<0.001	0.745
Locality	3, 36	3.01	0.042	
Cumulative °C × Locality	3, 36	7.28	<0.001	

**Table 3:** Above, GLM results showing the effects of sampling date (or cumulative °C) × year × locality on the number of CGW individuals per trap and day. Below, GLM results showing the effects of year × cumulative °C + locality × cumulative °C on the number of CGW individuals per trap and year.

Explanatory Variable	Individuals/Trap/Day			
	*df*	*F*	*p*	*R* ^2^
Year	3, 314	1.61	0.18	0.207
Locality	5, 314	5.39	<0.001	
Date	1, 314	0.77	0.37	
Year × Locality	15, 314	1.48	0.10	
Year × Date	3, 314	4.92	0.002	
Locality × Date	5, 314	0.52	0.75	
Year × Locality × Date	15, 314	0.85	0.62	
Year	3, 314	1.80	0.14	0.205
Locality	5, 314	4.88	<0.001	
Cumulative °C	1, 314	0.32	0.57	
Year × Locality	15, 314	1.30	0.19	
Year × Cumulative °C	3, 314	5.14	0.002	
Locality × Cumulative °C	5, 314	0.46	0.80	
Year × Locality ×Cumulative °C	15, 314	0.81	0.66	
**Individuals/Trap/Day**				
	***df***	***F***	***p***	***R*** ^**2**^
Year	3, 6	2.68	0.14	0.894
Locality	5, 6	5.23	0.033	
Cumulative °C	1, 6	0.00	0.99	
Year × Cumulative °C	3, 6	1.65	0.27	
Locality ×Cumulative °C	5, 6	1.95	0.21	

**Table 4:** Above, GLM results showing the effects of year × locality on the rate of sprouts with CGW galls and on the number of CGW galls per sprout. Below, GLM results showing the effects of year × annual mean temperature + locality annual mean temperature on the same damage measures. See significant post hoc differences in Table S3.

Explanatory Variable	Sprouts With Galls				Galls Per Sprout			
	*df*	*F*	*p*	*R* ^2^	*df*	*F*	*p*	*R* ^2^
Year	5, 182	14.80	<0.001	0.433	5, 182	18.50	<0.001	0.433
Locality	3, 182	7.35	0.001		3, 182	8.14	<0.001	
Year × Locality	9, 182	3.17	0.001		9, 182	1.36	0.20	
Year	5, 186	13.56	<0.001	0.371	5, 186	17.36	<0.001	0.405
Locality	3, 186	7.50	<0.001		3, 186	8.84	<0.001	
Annual mean temperature	1, 186	0.10	0.75		1, 186	0.72	0.39	
Year × Annual mean temperature	1, 186	1.70	0.19		1, 186	1.10	0.29	
Locality × Annual mean temperature	1, 186	0.60	0.40		1, 186	2.68	0.10	

## Data Availability

The data presented in this study are available on request from the corresponding author.

## Supplementary Materials

The following are available online at https://www.mdpi.com/article/10.3390/insects12100900/s1, Figure S1: Heatmap of locality per year clustered according to the values obtained for the average number of CGW individuals per development stage and the average survival probability per development stage. Yellow color denotes higher values, whereas red color denotes lower values; Figure S2: Heatmap of locality per year clustered according to the values obtained for CGW flight start date, flight peak date and flight end date. Yellow color denotes higher values, whereas red color denotes lower values; Figure S3: Heatmap of locality per year clustered according to the values obtained for the two CGW damage measures. Yellow color denotes higher values, red color denotes lower values; Table S1: Tukey’s HSD significant post hoc comparisons for the locality, locality × sampling date, locality × cumulative °C and season effects on CGW individuals per development stage and bud and on survival probability per stage; Table S2: Tukey’s HSD significant post hoc comparisons for the locality, year × sampling date and year × cumulative °C effects on CGW individuals per trap and day and on total CGW individuals per trap and year; Table S3: Tukey’s HSD pairwise significant post hoc comparisons for year, locality and their interactive effect on the assessed damage variables.
