# Supplementary figures and images for: Population Dynamics and Tree Damage of the Invasive Chestnut Gall Wasp, Dryocosmus kuriphilus, in Its Southernmost European Distributional Range
Source: Insects. 2021 Oct 2;12(10):900. doi: 10.3390/insects12100900 (PMC8540839; doi:10.3390/insects12100900)

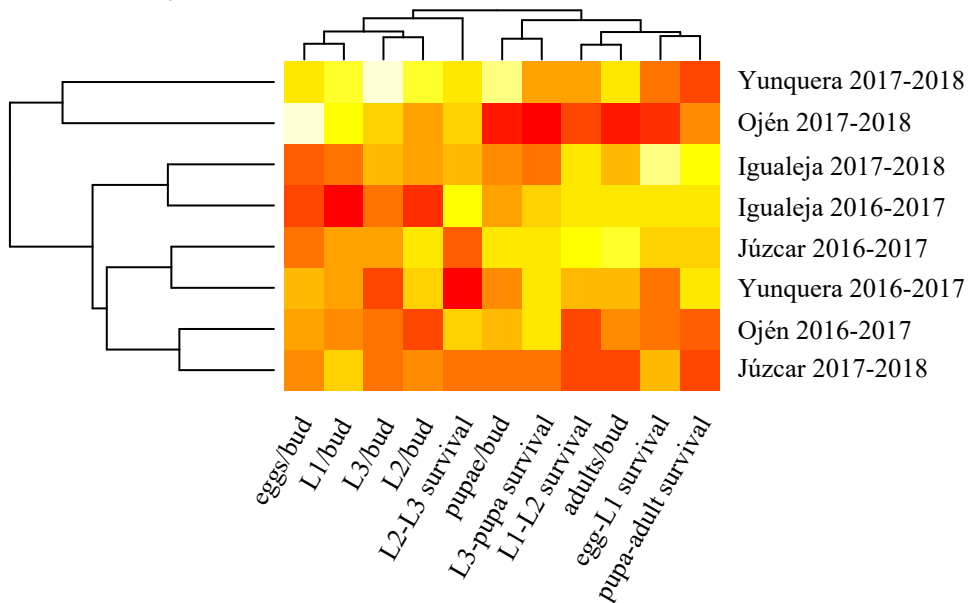

Supplement: Supplementary file 1 [file insects-12-00900-s001.zip › Supplementary materials/Figure S1.pdf]

# Color Key and Density Plot

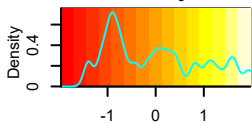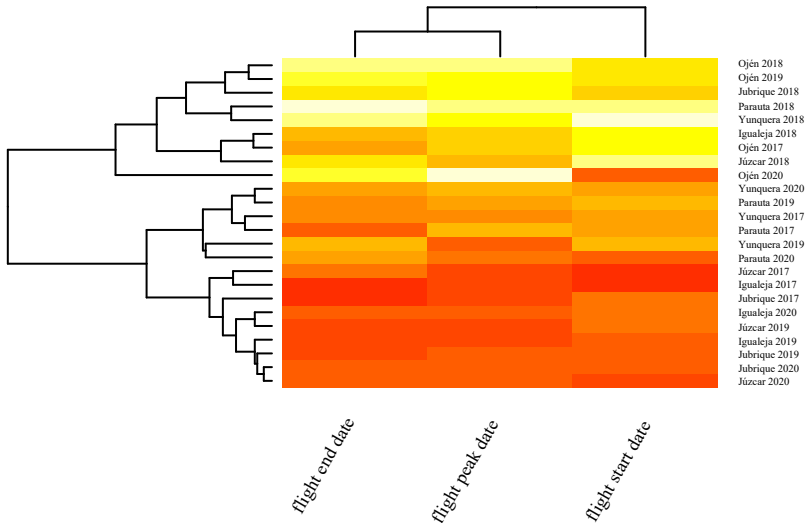

Supplement: Supplementary file 1 [file insects-12-00900-s001.zip › Supplementary materials/Figure S2.pdf]

Color Key  
and Density Plot

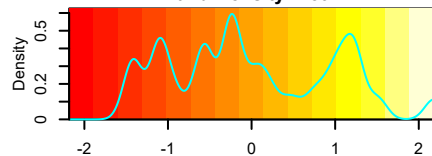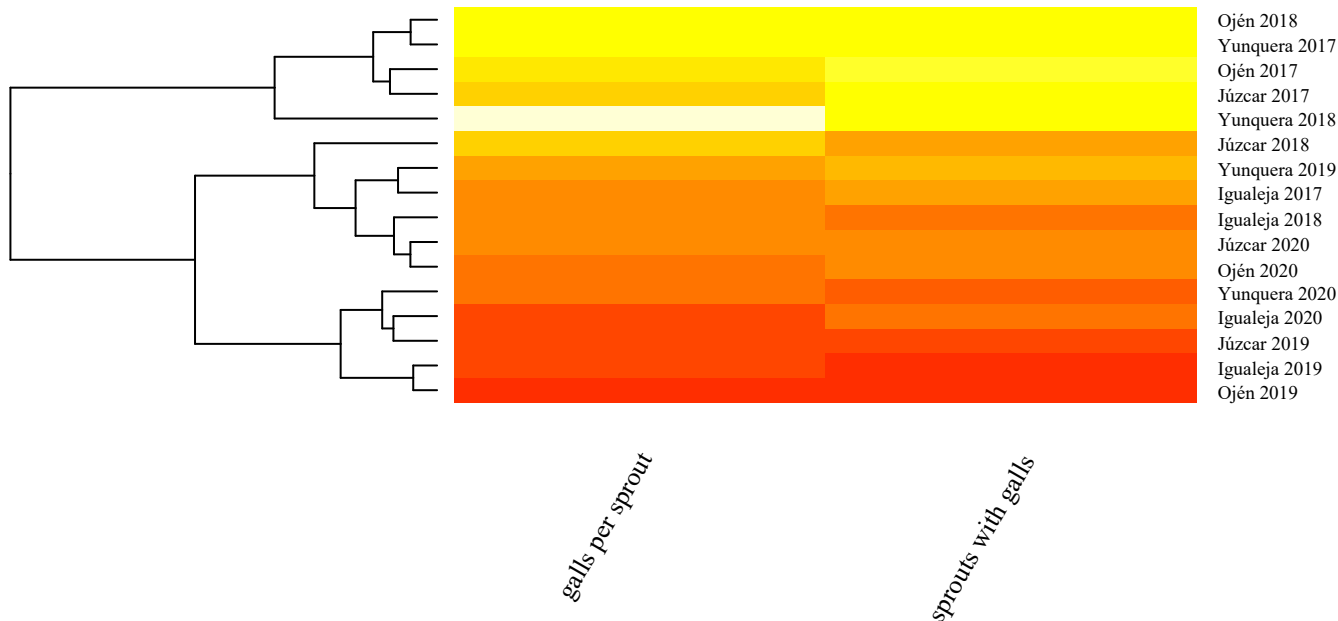

Supplement: Supplementary file 1 [file insects-12-00900-s001.zip › Supplementary materials/Figure S3.pdf]
